# Is daily computed tomography image guidance necessary for nasal cavity and nasopharyngeal radiotherapy? An investigation based on helical tomotherapy

**DOI:** 10.1120/jacmp.v9i1.2686

**Published:** 2008-01-28

**Authors:** Ke Sheng, Jennifer Chow, Grant Hunter, James Larner, Paul Read

**Affiliations:** ^1^ Department of Radiation Oncology University of Virginia Charlottesville Virginia U.S.A.

**Keywords:** Image‐guided radiation therapy, tomotherapy, setup errors, dosimetric error assessment

## Abstract

To analyze the magnitude of setup errors corrected by helical tomotherapy megavoltage computed tomography (MVCT) on a daily or weekly basis and the impact of those corrections on the delivered dose to the tumor and organs at risk (OARs), we retrospectively analyzed the setup errors for 6 nasal cavity and 4 nasopharyngeal cancer patients treated with helical tomotherapy for 25 – 33 fractions. Each patient had MVCT‐guided repositioning for all fractions of treatment. The new dose–volume histograms (DVHs) and equivalent uniform doses (EUDs) for the planning target volume (PTV) and OARs were calculated for hypothetical situations in which no imaging guidance (IG) or once‐weekly imaging guidance (WIG) took place. The mean total setup error for treatment without daily IG was 3.6±1.0 mm, which could be reduced to 1.7±0.6 mm if WIG were to be performed. The geometric uncertainties from the absence of IG resulted in a reduction of mean PTV EUD by 2.1%±1.0%, which could be reduced to 1.4%±1.0% with WIG. The EUDs of the OARs increased to 1.8±2.0 Gy or 0.8±1.3 Gy without and with WIG respectively. Without daily IG, the mean uncertainty in patient position has a relatively small effect on the mean dosimetry for PTV and OARs, and the use of WIG can further reduce those effects by approximately half. On the other hand, because of the large variance, with low probability, substantial deviation from the original planned dosimetry may occur without IG. Therefore, daily MVCT is preferred as an important safety measure in the delivery of intensity‐modulated radiation therapy.

PACS number: 87.53.Dq

## I. INTRODUCTION

Intensity‐modulated radiation therapy (IMRT) delivers radiation with greater conformality and minimizes dose to surrounding organs at risk (OARs), resulting in reduced toxicity. The IMRT technique is particularly advantageous in the treatment of head‐and‐neck cancer, in which multiple OARs are positioned in close proximity to the gross tumor volume (GTV); to the clinical target volume (CTV), which includes expansion for microscopic spread of the primary tumor and at‐risk lymph node volumes; and to the respective planning target volume (PTV), which includes an expansion for setup error and target and patient motion.^(^ ^1^ ^–^ ^6^ ^)^

However, the toxicity reduction in head‐and‐neck cancer cases treated with IMRT from highly conformal radiation isodose plans depends on steep isodose gradients to limit the dose to multiple adjacent surrounding OARs, and accurate treatment requires precise daily patient setup and rigorous immobilization. With standard head‐and‐neck radiotherapy immobilization techniques (conventional thermoplastic masks, baseplate fixation, three‐point laser alignment, and weekly portal film evaluation), a setup variability of 2 – 5 mm has been suggested by previous studies.^(^ ^7^ ^–^ ^9^ ^)^ Systematic errors of 1.6−4.6 mm and random errors of 1.1−2.5 mm were reported by Hurkmans et al. for portal imaging, with “state of the art” setup accuracy of 2 mm (one‐dimensional) standard deviation for both types of errors.^(8)^ A recent study by Hong et al. using a high‐precision optically‐guided patient localization system reported a 3.33 mm absolute average daily setup error in any one of three dimensions, with a composite vector setup error of 6.97 mm in three‐dimensional (3D) space.^(9)^

With the tighter treatment margins used in IMRT as compared with conventional radiotherapy techniques, these daily setup variations imply a theoretically higher risk of treatment effects by underdosing the primary tumor and nodal volumes if an inadequate PTV expansion is used and by overdosing adjacent normal structures, especially if systematic errors are present.^(^ ^10^ ^,^ ^11^ ^)^ For example, van Herk et al. determined that, in the context of accounting for random and systematic errors in conformal radiotherapy, aggressive reduction in treatment margins could lead to a significant loss of tumor control probability in prostate cancer treatment.^(12)^ In IMRT planning for head and neck cancer, in which steep dose gradients are required to spare adjacent critical structures, several authors have examined the potential effects of setup variability on equivalent uniform dose (EUD) and mean dose to the parotid gland,^(10)^ isodose distributions,^(13)^ and the dose–volume histogram (DVH).^(9)^ Ploquin et al. assessed the impact of systematic (variable from 0 mm to 6 mm) and random (fixed at 2 mm) setup uncertainties on treatment delivery compliance of oropharyngeal cancer IMRT planned with the Radiation Therapy Oncology Group H‐0022 treatment planning criteria using a Monte Carlo– based direct simulation approach. The DVHs and EUD were both used to describe the dose distributions achieved with various systematic shifts, and the authors concluded that, although most OARs meet dose constraints with up to 4 mm systematic uncertainty, systematic setup uncertainties greater than 2 mm could significantly compromise the dosimetry of the GTV and grossly involved lymph nodes.^(14)^

We previously showed improved conformality and dose homogeneity for helical tomotherapy (HT)–based IMRT as compared with 7‐field coplanar linear accelerator–based IMRT for oropharyngeal cancer.^(15)^ We showed equivalent OAR avoidance and improved homogeneity for HT‐based IMRT as compared with non‐coplanar linear accelerator–based IMRT for nasal cavity and paranasal sinus tumors.^(16)^

Historically, verification of treatment delivery accuracy has included port films or, more recently, electronic portal images obtained weekly—that is, in 20% of treatments. Newer linear accelerators and HT are capable of more advanced image‐guided radiation therapy (IGRT) by obtaining kilovoltage (kVCT) or megavoltage computed tomography (MVCT) scans on a daily basis and co‐registering them to the initial planning kVCT scans to improve daily patient positioning before treatment. To date, little work has been published regarding the potential benefit of daily IGRT for head‐and‐neck cancer patients or the optimal frequency of such imaging.^(^ ^11^ ^,^ ^17^ ^)^ Head‐and‐neck cancer patients treated at our institution with HT have typically undergone daily MVCT imaging (that is, 100% of treatments are image‐guided), with daily shifting according to deviations in the lateral, longitudinal, and anterior–posterior (AP) dimensions and in the degree of roll. In the present report, we made an effort to determine the required frequency of IGRT by retrospectively assessing the effect of image‐guided radiation therapy with daily imaging on the delivered dose, comparing the dose distributions that would have been obtained if no daily shifts had been made to the doses obtained by shifting the patient based on daily and weekly IGRT. Specifically, we retrospectively reviewed 10 nasal cavity and paranasal sinus and nasopharyngeal patients treated with HT, comparing the DVHs that incorporated daily shifts (“daily‐shifted DVH”), weekly shifts (“weekly‐shifted DVH”), and no shifts (“non‐shifted DVH”) with the planned DVHs. We determined the difference between daily‐shifted, weekly‐shifted, and non‐shifted dosimetry in mean highest 1% dose and EUD for the surrounding OARs and the PTV to determine the benefit of daily kVCT or MVCT co‐registration–based IGRT with respect to

- the accuracy of laser‐guided, Aquaplast (WFR/Aquaplast and Qfix Systems, Lawlins Park, Wyckoff, NJ)–immobilized head‐and‐neck patient setup positioning along the Cartesian axes (lateral, longitudinal, AP) and degree of roll.
- the effect of IGRT on setup error if daily, weekly, or no MVCT were used.
- the dosimetric effect in each patient's dose distribution profile.

## II. PATIENTS AND METHODS

The present retrospective review considers 10 patients with representative early and advanced nasal cavity and paranasal sinus and nasopharyngeal malignancies treated with HT‐based IMRT. Table 1 shows the clinical patient data. Most of the patients with nasal cavity and paranasal sinus tumors were treated preoperatively to 50 Gy per institutional protocol.

**Table 1:** Characteristics of 10 nasal cavity and nasopharyngeal cancer patients

Pt.	Age (years)	Sex	Primary site	Histology	Stage	Total dose(Gy)/total fractions (*n*)	Boost with tomotherapy [total dose (Gy)/fractions (*n*)]
1	14	M	Nasopharynx	Lymphoepithelial carcinoma	T1N1M0 (IIB)	56/28	14/7
2	75	F	Nasal cavity	Undifferentiated carcinoma	Kadish C	61.6/28	14/7
3	53	F	Nasopharynx	Low‐grade papillary adenocarcinoma	T1N0M0 (I)	66/30	
4	66	F	Nasopharynx	Adenoid cystic carcinoma	T4N0M0 (IV)	56/28	14/7
5	54	M	Nasopharyngeal with extension to base of skull	Undifferentiated carcinoma	T4N0M0 (IV)	39.6/22^a^	20/10
6	57	M	Nasal cavity	Esthesioneuroblastoma	Kadish B	50/25	
7	52	M	Nasal cavity	Poorly differentiated squamous cell carcinoma	T4bN0M0 (IVB)	50.4/28	
8	31	M	Infratemporal fossa	Rhabdomyosarcoma, alveolar type	Group 3 (III)	50.4/28	
9	64	F	Nasal cavity	Esthesioneuroblastoma	Kadish C	54/27	
10	59	M	Maxillary sinus	Poorly differentiated squamous cell carcinoma	T4N1M0 (IVB)	50/25	20/10

a Patient was re‐simulated after initial three treatments.

Pt.=patient; M=male; F=female.

### A. Patient planning and setup

Patients were set up in a MedTec S‐plate head‐and‐neck Aquaplast mask (MedTec, Orange City, IA) and CT‐simulated on a Philips AcQsim CT simulator with contiguous slices 3 mm thick. For all patients, the GTV was initially contoured to cover all gross disease, and a CTV was then contoured to cover microscopic spread of disease at the skull base, surrounding sinuses, and adjacent soft tissues. The CTV was customized based on the anatomic extent of the tumor, but for sinus tumors, it included the ipsilateral medial orbital wall, the nasal cavity, cribriform plate, bilateral ethmoid sinuses, and the ipsilateral maxillary and frontal osteomeatal complexes at a minimum, with larger expansions depending on tumor location and extent. The CTV included the entire nasopharynx, parapharyngeal space, and skull base with adequate margin for nasopharyngeal cancers. For appropriate cases, regional lymphatics at risk for microscopic spread were included in a second CTV (CTV2). To obtain the PTV (and PTV2, when appropriate), the CTV and CTV2 were expanded by 3 mm in all dimensions to account for patient setup error and motion within the Aquaplast mask. The expansions from CTV2 to PTV2 were limited to 5 – 7 mm from the skin surface to avoid overdosing the skin, with resultant moist desquamation. Normal critical structures included the brainstem, eyes, lens, optic nerves, optic chiasm, and parotid glands. Patient CT images and contours were then transferred from the planning system to the tomotherapy system. Plans were optimized so that the maximum dose to the optic structures, parotids, spinal cord, and brainstem would be as low as possible while a minimum of 95% of the PTV would be covered with the prescribed dose.

During HT treatments, patients were immobilized using standard head‐and‐neck immobilization techniques (head‐and‐shoulder Aquaplast masks, baseplate fixation) and underwent daily MVCT scans, which were co‐registered to the initial planning kVCT The image resolution of the MVCT scan is 0.78×0.78 mm with a slice thickness of 4 mm. Patients were repositioned according to co‐registration shifts in the lateral, longitudinal, and AP dimensions, and the HT gantry was repositioned to account for any roll shifts greater than 1 degree. The co‐registration was performed using mutual information. The combined uncertainty had been studied previously by Boswell et al.^(1)^ and found to be generally less than 1 mm in all directions for a head‐and‐neck phantom.

Setup errors were divided into two categories: systematic and random errors. Systematic errors are persistent displacements present throughout the entire course of fractionated therapy; random errors vary on a day‐to‐day basis.^(8)^ For individual patients, we evaluated for the presence of systematic error by averaging the daily shifts throughout the treatment course in each of the four degrees of freedom (lateral, longitudinal, and AP dimensions; degree of roll). Group systematic error was calculated by averaging the displacements for all 10 patients in each of the dimensions. A composite vector of deviations in the three Cartesian dimensions was calculated for each patient to reflect setup error in 3D space. The formula used was

(1) $$d_{3D}=\sqrt{d_{\text{lateral}}^{\;2}+d_{\text{longitudinal}}^{\;2}+d_{A-P}^{\;2}},$$

where $d_{\text{lateral}},d_{\text{longitudinal}},\;\text{and}\;d_{A-P}$ are deviations in the lateral, longitudinal, and AP directions respectively.

### B. Setup error with weekly CT and no image guidance

In making daily IGRT our clinical standard of care, we made the assumption that with daily IGRT, the therapists are able to perform the most accurate daily patient setup possible. For the purpose of the present study, we assumed that image guidance provided a perfect daily adjustment. Thus, zero deviation of patient treatment and planning position would be present after the adjustments were made. This assumption does not take into account image registration uncertainties or the fact that patients may not be perfectly aligned at all CT slices.

To calculate the degree of deviation if weekly, instead of daily, MVCT were to be used, the patient positioning was adjusted on the first fraction of the week. Consequently, the adjustment was subtracted from daily deviations as the new positioning error with weekly image guidance.

To meaningfully compare the setup error without image guidance and with weekly and daily guidance, the variance of shifts in the Cartesian dimensions and roll were calculated for weekly and for no MVCT for each patient. The equations used were

(2) $$\sigma_{\text{weekly}}=\sqrt{{\left(\vec{\delta }-{\vec{\delta }}_{w}\right)}^{2}/(N-1)}\;\;\;\text{and}$$

(3) $$\sigma_{\text{unregistered}}=\sqrt{\left({\vec{\delta }}^{2}/(N-1\right)},$$

where $\sigma_{\text{weekly}}$ and $\sigma_{\text{unregistered}}$ represent the standard deviations for weekly and no registration respectively, $\vec{\delta }$ is the daily translational shift, *N* is the total number of fractions, and ${\vec{\delta }}_{w}$ is the shift adjust for a weekly MVCT hypothetically performed at the first day of a week:

(4) $${\vec{\delta }}_{w}={\vec{\delta }}_{k},k=1,6,11\ldots 5n+1\ldots \;{\;}_{.}$$

### C. Obtaining dose distributions with and without daily shifts

Translational adjustments (in millimeters) along the Cartesian axes and degree of roll were obtained from the daily tomotherapy treatment delivery report, and the mean values of these shifts were calculated (Table 2). Degree of roll was not recorded for one patient. The dose was assumed to be invariant^(18)^ to displacement of the internal structures and of the whole patient. Mathematically, that assumption is expressed as

(5) $$D_{registered}(\vec{r})=\sum_{i=1}^{N}D_{0}(\vec{r}),$$

where $D_{0}$ is the planning dose for one fraction. However, if the daily shifts were not made following image registration, the new dose distribution for any given fraction becomes

(6) $$D_{1}(\vec{r})=rot(D_{0}(\vec{r}-\vec{\delta }),-\theta ),$$

where θ is the daily roll and *rot* is rotation of the dose matrix about the longitudinal axis. The cumulative dose was calculated using the expression

(7) $$D_{unregistered}(\vec{r})=\sum_{i=1}^{N}rot(D_{0}(\vec{r}-{\vec{\delta }}_{i}),-\theta_{i}),$$

where *N* is the total number of fractions.

**Table 2:** Mean value (standard deviation) of daily setup error for nasal cavity and nasopharyngeal cancer patients using megavoltage computed tomography (CT) co‐registration to initial planning kilovoltage CT

Pt.	Lateral (mm)	Longitudinal (mm)	AP (mm)	$d_{3D}$ (mm)	Roll (degree)
1	1.9 (0.9)	2.5 (5.0)	1.6 (0.7)	3.7 (4.7)	0.4 (0.4)
2	2.4 (1.1)	2.2 (1.6)	2.8 (1.9)	4.5 (1.5)	Not measured
3	1.2 (1.4)	2.7 (1.4)	2.3 (1.5)	4.0 (1.5)	1.0 (0.7)
4	2.3 (1.2)	3.8 (2.0)	2.5 (1.5)	5.3 (2.1)	0.3 (0.6)
5	2.5 (1.5)	2.6 (1.2)	1.8 (1.0)	4.0 (1.6)	0.7 (0.6)
6	1.8 (0.9)	1.5 (1.3)	0.9 (0.7)	2.8 (1.3)	0.3 (0.4)
7	0.6 (0.7)	2.7 (0.9)	1.2 (0.8)	3.0 (0.8)	0.4 (0.4)
8	2.3 (1.5)	2.0 (1.8)	2.1 (1.2)	4.1 (1.5)	1.7 (1.6)
9	0.8 (0.5)	1.3 (0.8)	0.7 (0.8)	1.9 (0.8)	1.7 (1.1)
10	1.1 (1.0)	1.3 (1.0)	1.6 (1.2)	2.7 (1.1)	0.2 (0.5)
Group	1.7 (1.1)	2.5 (1.7)	1.9 (1.1)	3.6 (0.9)	0.7 (0.7)

Pt.=patient; AP=anterior−posterior.

Result $D_{\text{unregistered}}(\vec{r})$ was then transferred to the ADAC ${\text{Pinnacle}}^{3}$ treatment planning system (Philips Medical Systems, Andover, MA) and superimposed onto the respective planning CT images and contours to calculate the cumulative DVHs of the PTV and surrounding structures. A similar calculation can also be performed on the recently available tomotherapy adaptive tool.

The total boost dose for four patients who received a boost on HT was added to their total primary treatment dose (Table 1), and the daily shifts made during the tomotherapy boost treatments contributed to the final dose distribution.

In the weekly image registration mode, one set of new surface marks per week was hypothetically made, and the patient would be set up following the new marks for that week. A similar operation was performed as outlined by Xia and colleagues^(4)^ and Gilbeau and colleagues^(7)^ to calculate $D_{weekly}(\vec{r})$ for each patient with the adjusted daily shifts. Three sets of $\text{DVHs}—{\text{DVH}}_{u}$ for $D_{\text{unregistered}}(\vec{r}),{\text{DVH}}_{r}$ for $D_{\text{registered}}(\vec{r})$, and ${\text{DVH}}_{w}$ for $D_{\text{weekly}}(\vec{r})$ —were then generated for comparison.

In the study, MVCT registration provided a patient daily shift that was incorporated into the DVH calculation without recalculation of the dose based on the partial volume acquired from the MVCT.

### E. EUD calculations

The EUDs were also calculated to provide an additional tool to quantitatively compare daily‐shifted, weekly‐shifted, and non‐shifted dose distributions. Niemierko first introduced the concept of EUD in 1997^(19)^ as the dose that, when delivered uniformly to the organ or target of interest, produces an effect similar to that of the non‐uniformly delivered dose:

(8) $$\text{EUD}={\left[(1/N_{v})\sum_{i}^{N_{v}}D_{i}^{a}\right]}^{1/a}.$$

In that equation, $N_{v}$ is the number of voxels in the anatomic structure of interest, $D_{i}$ is the dose in the *i* th voxel and *a* is the tumor‐ or normal tissue‐specific optimization parameter describing the dose‐volume effect. The *a* values used in our calculations, shown in Table 3, were based on values published by Wu et al.^(20)^ Because *a* values are not available for optic structures, we used an optimization parameter of 7.4 (similar to that for spinal cord) for eye and optic nerve calculations because of the character of those structures as serial organs with a radiation tolerance similar to that of spinal cord.

**Table 3:** The equivalent uniform dose a optimization parameters for the head‐and‐neck target and normal structures

	Target	Brainstem	Spinal cord	Parotids	Eye	Optic nerve
*a*	−8.0	4.6	7.4	5.0	7.4	7.4

Statistics were calculated using the *t*‐test function.

## III. RESULTS

The mean $d_{3D}$ calculated from equation 1 for all patients without image guidance was 3.6±1.0 mm, and the mean $d_{3D}$ for weekly image guidance was 1.7±0.6 mm. By using weekly MVCT imaging and registration, the setup error can be reduced by half (p=0.05).

**Figure 1:**
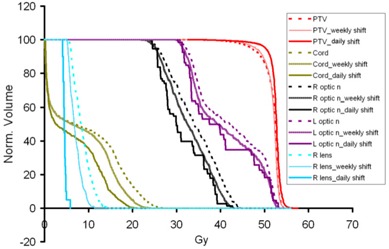
A typical dose–volume histogram comparison of selected organs for a patient in three scenarios: (1) daily repositioned with megavoltage computed tomography (MVCT) co‐registration with original planning computed tomography (CT) image; (2) with only weekly MVCT imaging guidance; and (3) without any imaging guidance, indicated by dotted, thin and thick solid lines respectively. Compared with scenario 1, scenario 3 shows slightly degraded dosimetry, with additional underdosed area in the planning target volume (PTV) and overdosed area in most organs at risk. Scenario 2 falls between scenarios 1 and 3.

Fig. 1 shows the effect of the setup error on the three types of DVHs for a typical patient. The quality of dose coverage for the PTV degraded slightly, dose to OARs increased slightly, and the PTV contained cooler spots if no daily image guidance was performed. The ${\text{DVH}}_{w}$ for weekly imaging guidance falls between the ${\text{DVH}}_{r}$ and the ${\text{DVH}}_{u}$.

**Figure 2:**
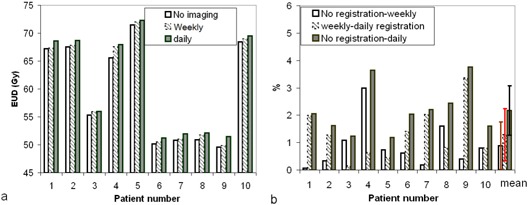
The lowered equivalent uniform dose (EUD) in the planning target volume attributable to the lack of daily image guidance. (a) Absolute values of EUD. (b) Normalized values of EUD.

The qualitative observations from these DVH differences were further analyzed by reviewing the EUD of the PTV. From Fig 2(a), the PTV EUD was lowered for all patients without daily image registration, and weekly registration improved the EUD. Because patients had different total prescribed doses, the magnitude of the improvement was more evident when presented as a relative comparison, as in Fig. 2(b), which shows that the EUD of the PTV was reduced by an average of 2.1%±1.0% if no image guidance was used, as compared with 1.3% 1.3%±1.0% with weekly image guidance. In other words, by using weekly registration, the total number of MVCT images can be reduced by a factor of 5, and 40% of the potential EUD loss to the PTV can be recovered.

To analyze the dosimetric impact from the lack of image guidance, we applied the EUD calculation to the OARs. Because of the substantial differences in the planning dose, the EUD comparison is presented as relative differences in Gy. Table 4 shows the mean and standard deviation for EUD errors of OARs. Without image registration, the EUD to surrounding OARs did not change substantially—in part because of good patient immobilization and laser localization system. The results from the 10 study patients show only an overall trend of OAR EUD increment of approximately 1.8±2.0 Gy. Because of the large variation, the result is not statistically significant (p=0.31 by *t*‐test). With weekly image guidance and patient re‐positioning, the OAR EUD error is reduced by approximately 1.5 Gy to 0.8±1.3 Gy, a more effective reduction than that of PTV EUD error. The EUDs to OARs were within tolerance in the original plan. The slight increase in dose because of the removal of imaging guidance did not result in the tolerance being exceeded.

**Table 4:** The change (standard deviation) in equivalent uniform dose (EUD, in Grays) caused by daily positioning error for organs at risk

	Eyes	Spinal cord	Parotids	Brainstem	Optic chiasm	Optic nerves
${\text{EUD}}_{\text{no}\;\text{registration}}-{\text{EUD}}_{\text{weekly}}$	1.7 (1.6)	1.1 (1.4)	1.1 (1.5)	0.8 (0.9)	1.6 (2.0)	1.2 (0.9)
${\text{EUD}}_{\text{weekly}}-{\text{EUD}}_{\text{daily}}$	0.2 (1.0)	1.4 (1.6)	1.0 (1.3)	0.8 (1.1)	0.3 (1.2)	−0.6 (1.1)
${\text{EUD}}_{\text{no}\;\text{registration}}-{\text{EUD}}_{\text{daily}}$	1.9 (2.0)	2.5 (2.7)	2.1 (1.5)	1.6 (1.6)	1.9 (1.7)	0.6 (1.5)

## IV. DISCUSSION

Head‐and‐neck patients with Aquaplast masks are reasonably well immobilized with an average setup uncertainty of 3.6 mm (Table 2), which does not include registration uncertainties (which have been shown to be generally less than 1 mm for a head‐and‐neck phantom,^(21)^ with a slightly larger error in the longitudinal direction). The subvoxel registration accuracy is achievable because the registration is based on a large number of voxels. Thévenaz and Unser^(22)^ reported one application that uses the mutual information algorithm to attain subpixel accuracy. Assuming that the registration error is 1 mm and independent of the patient setup error, and using the error propagation formula that total uncertainty is the root sum square of individual uncertainties, the patient average setup error is reduced to 3.5 mm from 3.6 mm. Therefore, the registration uncertainty should not affect the dose calculation.

**Figure 3:**
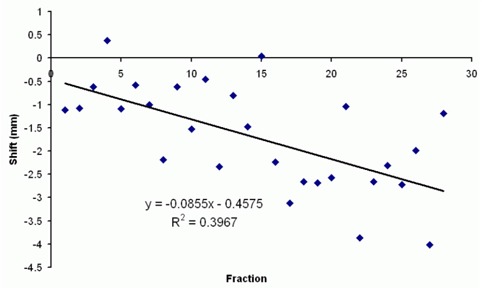
The vertical daily adjustment with megavoltage computed tomography of a patient shows a loose linear trend in one direction.

Two types of setup errors are involved: random and systematic. Both types are observed in the 10 study patients. Fig. 3, in which the daily registration value of lateral shift for 1 patient is charted for 35 fractions, shows examples of the two types of errors. Randomness is evident in the day‐to‐day variation; however, a trend of shifting toward a direction is also seen. That trend could be caused by progressive weight loss during the course of treatment: As the Aquaplast mask becomes loose, the patient may move toward one side of the mask out of preference. The slope of the linear regression ranged from 0 mm to 0.15 mm per fraction in 3 directions among the 10 study patients. However, because of the generally low $R^{2}$ value (<0.5), predicting the slopes based on a few initial images and using them as a predictive measurement of setup error for individual patients is difficult.

If the shift made by daily image registration is completely random, weekly image guidance would have little value in correcting setup error. However, because of the trend of shifting, the systematic components of setup error can be minimized by applying a weekly image registration. The effectiveness of weekly image guidance is reflected in both the reduction in geometric errors and in dosimetric errors.

**Figure 4:**
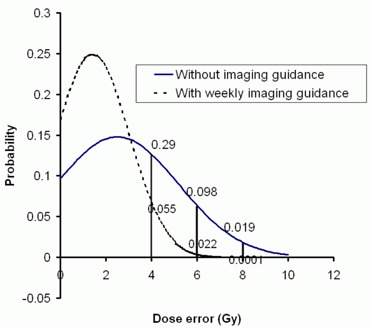
The normal distribution of spinal cord dose error from a treatment plan without and with weekly imaging guidance.

Given the cost of MVCT imaging dose to a large volume of the patient, the mean dosimetric error corrected by daily imaging may not be significant. However, considering the substantial deviation, gross dosimetric error may occur at low probability. Fig. 4 shows the normal distribution of the spinal cord position using the mean and deviation calculated in Table 2. The probability of a 6‐Gy or greater error can be observed to be 0.098 and of an 8‐Gy or greater error, 0.019. However, the corresponding probabilities of error can be reduced to 0.022 and 0 respectively if weekly or daily imaging are applied (assuming that the patient can be accurately positioned with MVCT). Similar results were observed with other critical organs. Therefore, the main function of daily imaging guidance may be its importance as a safety measure to prevent large deviations that could result in tragic and preventable complications in a small number of patients.

Because of the rapid dose falloff outside of the target volume, and therefore the higher dose gradient, nearby OARs were affected by the slight positioning error more than was the PTV, which is in a more homogeneous dose volume.

Intrafractional motion may happen after the MVCT scan and patient repositioning, although that motion is limited by the immobilization device. A real‐time monitoring device would be needed to study that type of motion, and its consideration is beyond the scope of this particular study.

## V. CONCLUSIONS

We studied the magnitude of daily patient positioning errors corrected by tomotherapy MVCT image registration. The composite geometric error of three Cartesian axes exceeded the PTV margin expansion of 3 mm. However, weekly image registration could reduce the error to 1.7±0.6 mm, which is well below the PTV margin used for the setup uncertainty. On the other hand, the increased geometric uncertainty resulted in only a 2% reduction of the PTV EUD, and half of the dose error could be recovered by weekly image guidance with improved clinic efficiency. The geometric uncertainty increased the OAR EUD dose by 1.8±0.6 Gy with large variation, which means that, in a worst‐case scenario for spinal cord, the EUD dose could have been increased by 6 Gy with 10% probability without image guidance. With weekly imaging, the worst case would result in an EUD error to the same organ of 3 Gy, mainly by reducing the systematic setup error. Therefore, daily image guidance has limited value in improving mean dose delivery accuracy in a collective patient population, but it has greater value in preventing large dosimetric errors in a small percentage of patients who experience additional systematic and random setup error. Because of a lack of predictive parameters to differentiate patient setup quality before treatment, we consider daily MVCT imaging guidance to be a preferable step and an important safety measure in IMRT treatment of head‐and‐neck cancer.

## Supporting information

Supplementary Material
