# Supplementary material for: Is daily computed tomography image guidance necessary for nasal cavity and nasopharyngeal radiotherapy? An investigation based on helical tomotherapy
Source: J Appl Clin Med Phys. 2008 Jan 28;9(1):36–46. doi: 10.1120/jacmp.v9i1.2686 (PMC5721532; doi:10.1120/jacmp.v9i1.2686)
Supplement: Supplementary file 1 — Supplementary Material [file ACM2-9-36-s001.doc]

**Is Daily CT image Guidance Necessary for Nasal Cavity and Nasopharyngeal Radiotherapy? An Investigation Based on Helical Tomotherapy**

Key words: Image guided radiation therapy, Tomotherapy, set up errors, dosimetric error assessment

# Abstract

In order to analyze the magnitude of set up errors corrected by Helical TomoTherapy Mega-Voltage CT on a daily or weekly basis and their impact on the delivered dose to the tumor and organs at risk (OAR), the set up errors of 6 nasal cavity and 4 nasopharyngeal cancer patients who were treated with Helical Tomotherapy for 25-33 fractions were retrospectively analyzed. Each patient had MVCT guided repositioning for all fractions of treatment. The new dose volume histogram (DVH) and equivalent uniform dose (EUD) for planning target volume (PTV) and OARs were calculated for hypothetical situations where no imaging guidance (IG) or once weekly image guidance (WIG) took place. The mean total set up error if treated without daily IG was 3.6±1.0 mm, which can be reduced to 1.7±0.6 mm if a WIG was performed. The geometrical uncertainties from the absence of image guidance resulted in a reduction of mean PTV EUD dose by 2.1±1.0%, which can be reduced to 1.4±1.0% with WIG. The EUD of OARs increased 1.8±2.0Gy or 0.8±1.3Gy without or with WIG respectively. Without daily IG, the mean patient position uncertainty has relatively small impacts on the mean PTV and OAR dosimetry, which can be further reduced approximately by half using a WIG. On the other hand, because of the large variance, with low probability, substantial deviation from the original planned dosimetry may occur without IG. Therefore, daily MVCT is preferred as an important safety measure in the IMRT.

# Introduction

The advent of intensity-modulated radiation therapy confers the ability to deliver radiation with greater conformality and to minimize dose to surrounding organs at risk (OARs) resulting in reduced toxicity. This is particularly advantageous when treating head and neck cancer, where multiple OARs are positioned in close proximity to the gross tumor volume (GTV), clinical target volume (CTV) which includes an expansion for microscopic spread of the primary tumor and at risk lymph node volumes, and its respective planning target volumes (PTV) which includes an expansion for set up error and target/patient motion [1-6].

However, the toxicity reduction in patients treated with IMRT for head and neck cancer from highly conformal radiation isodose plans depends on steep isodose gradients to limit the dose to multiple adjacent surrounding OARs, and accurate treatment requires precise daily patient set-up and rigorous immobilization. With standard head and neck radiotherapy immobilization techniques (conventional thermoplastic masks, baseplate fixation, three-point laser alignment, and weekly portal film evaluation), a set up variability of 2-5 mm has been suggested by previous studies [7-9]. Systematic errors ranging from 1.6-4.6 mm and random errors from 1.1-2.5 mm were reported by Hurkmans *et al* using portal imaging—with ‘state of the art’ set up accuracy of 2 mm (1D) standard deviation for both types of errors [8]. A recent study by Hong *et al* using a high-precision optically guided patient localization system reported a 3.33 mm absolute average daily set up error in any single one of three dimensions, with a composite vector set up error of 6.97 mm in 3D space [9].

With the tighter treatment margins used with IMRT compared to conventional radiotherapy techniques, these daily set up variations imply a theoretically higher risk of impacting treatment by under dosing the primary tumor and nodal volumes if an inadequate PTV expansion is used and overdosing adjacent normal structures, especially if systematic errors are present [11, 16]. For example, van Herk *et al* determined that in the context of accounting for random and systematic errors in conformal radiotherapy, aggressive reduction in treatment margins could lead to a significant loss of tumor control probability in prostate cancer treatment [10]. In IMRT planning for head and neck cancer, where steep dose gradients are required to spare adjacent critical structures, several authors have examined the potential effects of set up variability on equivalent uniform dose (EUD) and mean dose to the parotid gland [11], isodose distributions [12], and the DVH [9]. Ploquin *et al* assessed the impact of systematic (variable from 0 to 6 mm) and random (fixed at 2 mm) set up uncertainties on treatment delivery compliance of oropharyngeal cancer IMRT planned with the RTOG H-0022 treatment planning criteria using a Monte Carlo based direct simulation approach. Both DVHs and EUD were used to describe the dose distributions achieved with different systematic shifts and they concluded that while most OARs meet dose constraints with up to 4 mm systematic uncertainty, systematic set up uncertainties greater than 2 mm could significantly compromise the dosimetry of the GTV and grossly involved lymph nodes [13].

We have previously shown improved conformality and dose homogeneity for Helical Tomotherapy (HT)-based IMRT compared to seven field co-planar linear accelerator-based IMRT for oropharyngeal cancer [14]. We have shown equivalent OAR avoidance and improved homogeneity for HT-based IMRT compared to non-coplanar linear accelerator-based IMRT for nasal cavity and paranasal sinus tumors [15].

Historically, verification of treatment delivery accuracy has included port films or more recently electronic portal images obtained weekly, ie 20% of treatments. Newer linear accelerators and HT are capable of more advanced image guided radiation therapy (IGRT) by obtaining kVCT or MVCT scans on a daily basis and co-registering them to the initial planning kVCT scans to improve daily patient positioning prior to treatment. To date there is little published work regarding the potential benefit of such daily IGRT for head and neck cancer patients or the optimal frequency of such imaging [16-17]. Head and neck cancer patients treated at our institution with HT have undergone daily megavoltage CT imaging (ie 100% of treatments were image guided) and were shifted daily according to deviations in lateral, longitudinal, anterior-posterior (AP) dimensions, and degree of roll. In this report we retrospectively assessed the impact of image guided radiation therapy with daily imaging on the delivered dose by comparing the dose distributions that would have been obtained if no daily shifts were made and comparing them to the dose obtained by shifting the patient based on daily and weekly IGRT in an effort to determine the frequency of IGRT necessary. Specifically, we performed a retrospective review of 10 nasal cavity and paranasal sinus and nasopharyngeal patients treated on HT to determine the benefit of daily kVCT/MVCT co-registration based image guided radiation therapy with respect to: 1) the accuracy of laser guided, aquaplast immobilized H&N patient set up positioning along the Cartesian axes (lateral, longitudinal, AP dimensions), and degree of roll; 2) the impact of IGRT on set up error if daily, weekly or no MVCT were used; 3) the dosimetric impact in each patient’s dose distribution profile by comparing the DVH that incorporates daily shifts (“daily-shifted DVH”), weekly shifts (“weekly-shifted DVH”), and the DVH without shifts (“non-shifted DVH”) with the planned DVH; and the difference between daily-shifted, weekly-shifted, and non-shifted dosimetry in mean highest 1% dose and EUD for surrounding OARs and the PTV.

1. **Methods and Materials:**

This is a retrospective review of ten patients with representative early and advanced nasal cavity and paranasal sinus and nasopharyngeal malignancies treated with HT-based IMRT. The clinical patient data is shown in Table 1. Most of the nasal cavity and paranasal sinus tumor patients were treated preoperatively to 50 Gy as per institutional protocol.

*2.1. Patient planning and set up*

Patients were set up in a MedTec S-plate head and neck aquaplast mask (MedTec, Orange City, Iowa) and CT simulated on a Philips AcQsim® CT Simulator with contiguous 3 mm thick slices. The Gross Tumor Volume (GTV) was initially contoured to cover all gross disease and a Clinical Target Volume (CTV) was then contoured to cover microscopic spread of disease at the skull base, surrounding sinuses and adjacent soft tissues for all patients. The CTV was customized based on the anatomic extent of the tumor, but for sinus tumors included the ipsilateral medial orbital wall, the nasal cavity, cribiform plate, bilateral ethmoid sinuses, and the ipsilateral maxillary and frontal osteomeatal complexes at a minimum with larger expansions depending on tumor location and extent. The CTV included the entire nasopharynx, parapharyngeal space, and skull base with adequate margin for nasopharyngeal cancers. For appropriate cases regional lymphatics at risk for microscopic spread were included in a second CTV2. The CTV1 and CTV2 were expanded by 3 mm in all dimensions to account for patient set up error and motion within the aquaplast mask to obtain the Planning Target Volumes (PTV1 and PTV2 respectively). The expansions from CTV2 to PTV2 were limited to 5-7 mm from the skin surface to avoid overdosing the skin and resultant moist desquamation. Normal critical structures included the brainstem, eyes, lens, optic nerves, optic chiasm, and parotid glands. Patient CT images and contours were then transferred from the planning system to the Tomotherapy system. Plans were optimized to limit the maximum dose to the optic structures, parotids, spinal cord, and brainstem to as low as possible while covering a minimum of 95% of the PTV with the prescribed dose.

During HT treatments, patients were immobilized with standard head and neck immobilization techniques (head and shoulder aquaplast masks, baseplate fixation), and underwent daily MVCT scans, which were co-registered to the initial planning kVCT. The image resolution of the MVCT scan is 0.78 mm × 0.78 mm with a slice thickness of 4 mm. Patients were repositioned according to co-registration shifts in the lateral, longitudinal, AP dimensions and the HT gantry was repositioned to account for any roll shifts greater than one degree. The co-registration was performed using mutual information (MI). The combined uncertainty was previous studied by Boswell et al [1] and found to be generally less than 1 mm in all directions for a head-and-neck phantom.

Set up errors were divided into two categories: systematic and random errors. Systematic errors are persistent displacements present throughout the entire course of fractionated therapy while random errors vary on a day-to-day basis [8]. For individual patients, we evaluated for the presence of systematic error by averaging the daily shifts throughout the treatment course in each of the four degrees of freedom (lateral, longitudinal, AP dimensions, degree of roll). Group systematic error was calculated by averaging all ten patients’ displacements in each of the dimensions. A composite vector of deviations in three Cartesian dimensions was calculated for each patient to reflect set up error in 3D space using the formula:

$\text{d}_{\text{3D }}\text{= }\sqrt{\text{d}^{2}_{\text{lateral }}\text{+ d}^{2}_{\text{longitudinal}}\text{ + d}^{2}_{\text{A-P}}}$*,* (1)

where *d*_lateral_, *d*_longitudinal_, and *d*_A-P_ are deviations in lateral, longitudinal, and anterior-posterior directions, respectively.

*2.2. Set up error with weekly CT and no image guidance*

In making daily IGRT our clinical standard of care, we made the assumption that with daily IGRT, the therapists were able to perform the most accurate daily patient set up possible. For the purpose of this study we assumed that image guidance provided a perfect daily adjustment; thus there would be zero deviation of patient treatment and planning position after the adjustments were made. This assumption does not take into account image registration uncertainties or the fact that patients may not be perfectly aligned at all CT slices.

To calculate the degree of deviations if weekly, instead of daily MVCT was used, the patient positioning was adjusted on the first fraction of the week. Consequently, the adjustment was subtracted from daily deviations as the new positioning error with weekly image guidance.

In order to meaningfully compare the set up error with weekly, daily and without image guidance, the variance of shifts in the Cartesian dimensions and roll were calculated for weekly and no MVCT for each patient according to the following equations:

$\sigma_{\text{weekly}}\text{ = }\sqrt{\text{(}\vec{\delta}\text{ - }\vec{\delta}_{\text{w}}\text{)}^{2}\text{/(N - 1)}}\text{ }$ (2)

$\sigma_{\text{unregistered}}\text{ = }\sqrt{\vec{\delta}^{2}\text{/(N- 1)}}$ (3)

Where $\sigma_{\text{weekly}}$ and $\sigma_{\text{unregistered}}$ represents standard deviations for weekly and without registration, respectively, $\vec{\delta}$ is the daily translational shift, *N* represents the total number of fractions, $\vec{\delta}_{\text{w}}$ is the shift adjust for a weekly MVCT hypothetically performed at the first day of a week,

$\vec{\delta}_{\text{w}}=\vec{\delta}_{k},k=1,6,11...5n+1...$ (4)

## 2.3. Obtaining dose distributions with and without daily shifts

Translational adjustments (mm) along the Cartesian axes and degree of roll were obtained from the daily Tomotherapy treatment delivery report and the mean values of these shifts were calculated and are shown in Table 2. One patient did not have degree of roll recorded. The dose was assumed to be invariant (21) to displacement of the internal

structures and of the whole patient. Mathematically,

$D_{registered}(\vec{r})=\sum_{i=1}^{N} D_{0}(\vec{r})$, (5)

where$D_{0}$is the planning dose for one fraction. However, if the daily shifts were not made following the image registration, the new dose distribution for any given fraction becomes

$D_{1}(\vec{r})=rot(D_{0}(\vec{r}-\vec{\delta}),-\theta)$, (6)

where$\theta$ is the daily roll and *rot* is rotation of the dose matrix about the longitudinal axis. The cumulative dose is calculated by:

$D_{unregistered}(\vec{r})=\sum_{i=1}^{N} rot(D_{0}(\vec{r}-\vec{\delta}_{i}),-\theta_{i})$, (7)

given the total number of fractions is *N*.

$D_{unregistered}(\vec{r})$ was then transferred to ADAC Pinnacle^3^ Treatment Planning System® (Philips Medical Systems, MA) and superimposed onto the respective planning CT images and contours to calculate the cumulative DVHs of the PTV and surrounding structures. The similar calculation can also be performed on the recently available Tomotherapy adaptive tool.

The total boost dose for four patients who received a boost on HT was added to their total primary treatment dose (Table 1), and the daily shifts made during the Tomotherapy boost treatments contributed to the final dose distribution.

In the weekly image registration mode, one set of new surface marks is made hypothetically per week and the patient would be set up following the new marks for that week. A similar operation was performed as outlined by (4) and (7) to calculate $D_{weekly}(\vec{r})$ for each patient with the adjusted daily shifts.

Three sets of DVHs, DVH_u_ for$D_{unregistered}(\vec{r})$, DVH_r_ for$D_{registered}(\vec{r})$ and DVH_w_ for $D_{weekly}(\vec{r})$, were then generated for comparison.

### In the study, MVCT registration provided a patient daily shift, which was incorporated into the DVH calculation without re-calculate the dose based on the partial volume acquired from the MVCT.

### *2.5 EUD calculations*

EUD was also calculated to provide an additional tool to quantitatively compare daily-shifted, weekly-shifted, and non-shifted dose distributions. Niemierko first introduced the concept of equivalent uniform dose in 1997 as the dose, when delivered uniformly to the organ or target of interest, will produce similar effect as the non-uniformly delivered dose (18).

$\text{EUD = [(1/N}_{\text{v}}\text{) }\sum_{\text{i}}^{\text{N}_{\text{v}}} \text{D}_{\text{i}}^{\text{a}}\text{ ]}^{\text{1/a}}$ (8)

In this equation, *N_v_* is the number of voxels in the anatomic structure of interest, *D_i_* is the dose in the *i*’th voxel and *a* is the tumor or normal tissue-specific optimization parameter describing the dose-volume effect. The *a* values used in our calculations, shown in Table 3 are based on the values published by Wu *et al* [19]. Since *a* values are not available for optic structures, an optimization parameter of 7.4, similar to that of spinal cord, was used for eye and optic nerve calculations because of their similarity as serial organs and similar radiation tolerance.

The statistic study was calculated using t-test function.

**3. Results:**

The mean d_3D_ calculated from equation (1) for all patients without image guidance is 3.6 ±1.0 mm, and the mean d_3D_ for weekly image guidance is 1.7±0.6 mm. By using weekly MVCT imaging and registration, the set up error can be reduced by half (p=0.05). The impact of the set up error on the DVH for a typical patient is shown in Fig. 1

Fig. 1 shows the three types of DVHs of one patient. The quality of dose coverage for the PTV degraded slightly, dose to organs at risk increased slightly and the PTV contained cooler spots if no daily image guidance was performed. The DVH_w_ for weekly imaging guidance falls in between DVH_r_ and DVH_u_.

The qualitative observations from these DVH differences were further analyzed by EUD of the PTV. From Fig 2 (a), for all patients the PTV EUD was lowered without daily imaging registration and the weekly registration improved the EUD. Because patients had different total prescribed doses, the magnitude of improvement is more evident when presented as a relative comparison exhibited in Fig. 2 (b), which shows that the EUD of the PTV was reduced by an average of 2.1±1.0% if no image guidance was used, compared with a 1.3±1.0% reduction with weekly image guidance. In other words, by using weekly registration, the total number of MVCT images can be reduced by five fold and 40% of the potential EUD loss to the PTV can be recovered.

The EUD calculation was applied to organs at risk (OARs) to analyze the dosimetric impact from the lack of image guidance. Because of the substantial difference in their planning dose, the EUD comparison is presented as their relative differences in Gy. Table 4 shows the mean EUD errors of OARs and standard deviation. The EUD to surrounding OARs did not change substantially without image registration in part due to good patient immobilization and laser localization system. The results from these 10 patients only show an overall trend of OAR EUD increment of approximately 1.8±2.0 Gy. Because of the large variation, the result is not statistically significant (p=0.31 using t-test). With weekly image guidance and patient re-positioning, the OAR EUD error is approximately reduced by 1.5 Gy to 0.8 ±1.3 Gy, a more effective reduction than that of PTV EUD error. The EUDs to OARs were within the tolerance in the original plan. The slight increase in dose due to the removal of imaging guidance did not result in exceeding the tolerance.

**4. Discussion:**

Head and neck patients with aquaplast masks are reasonably well immobilized with an average set up uncertainty of 3.6 mm (table 2), which does not include the registration uncertainties that was shown to be general less than 1 mm for a head and neck phantom[20] with the slightly larger error in the longitudinal direction. The sub-voxel registration accuracy is achievable because the registration is based on a large number of voxels. One application of using the mutual information algorithm to attain sub-pixel accuracy was reported by Thevenaz and Unser [22]. Assuming the registration error is 1 mm and independent to the patient set up error, and using the error propagation formula that the total uncertainty is the root sum square of individual uncertainties, the patient average set up error is reduced to 3.5 mm from 3.6 mm. Therefore, the registration uncertainty should not affect the dose calculation.

There are two types of set up errors involved: random and systematic. They are both observed in our 10 patients. Examples of the two types of errors are shown in Fig. 3 where the daily registration value of lateral shift for one patient is charted for 35 fractions. There is evident randomness exhibited in the day to day variation, however, there is also a trend of shifting toward a direction. This could be caused by progressive weight loss during the course of treatment and as the aquaplast mask becomes loose, the patient may move towards one side of the mask out of preference. The slope of the linear regression ranged from 0 to 0.15 mm/fraction among the 10 patients in 3 directions. However, because of the generally low R^2^ value (<0.5), it is difficult to predict the slopes based on a few initial images and use these as a predictive measurement of set up error for individual patients.

If the shift made by daily image registration is completely random, weekly image guidance would have very little value in correcting set up error. However, because of the trend of shifting, by applying a weekly image registration, the systematic components of set up error can be minimized. The effectiveness of weekly image guidance is reflected in both the reduction of geometrical errors and dosimetric errors.

The mean dosimetric error corrected by daily imaging may not be significant at a cost of MVCT imaging dose to a large volume of the patient. However, considering the substantial deviation, gross dosimetric error may occur at low probability. Fig. 4 shows the normal distribution of the spinal cord position using the mean and deviation calculated in table 2. It can be observed that the probability of having 6 Gy or higher error is 0.098 and 8 Gy or higher is 0.019. However, the correspondent probabilities of errors can be reduced to 0.022 and 0 respectively if weekly or daily imaging was applied and assuming that patient can be accurately positioned with MVCT; similar results were observed with other critical organs. Therefore, the main function of daily imaging guidance may be important as a safety measure to prevent large deviations in a small number of patients that could result in tragic and preventable complications.

Because of the rapid dose fall off outside of the target volume and therefore higher dose gradient, nearby OARs were affected by the slight positioning error more than the PTV, which is in a more homogeneous dose volume.

Intra-fractional motion may happen after the MVCT scan and patient repositioning, although the motion is limited by the immobilization device. A real time monitoring device would be needed to study this type of motion and is beyond the scope of this particular study.

**5. Conclusions:**

In this paper, we studied the magnitude of daily patient positioning errors corrected by the TomoTherapy MVCT image registration. The composite geometric error of three Cartesian axes exceeds the PTV margin expansion of 3 mm. However, weekly image registration can reduce the error to 1.7 ±0.6mm, which is well below the PTV margin used for the set up uncertainty. On the other hand, the increased geometric uncertainty resulted in only a 2% decrease of the PTV EUD, and half of the dose error can be recovered by weekly image guidance with improved clinic efficiency. The geometric uncertainty increased the OAR EUD dose by 1.8±0.6 Gy with large variation, which means, in a worst case scenario, for spinal cord, the EUD dose could have been increased by 6 Gy with 10% probability without image guidance. With weekly imaging, the worst case would result in a 3 Gy EUD error to the same organ by mainly reducing the systematic set up error. Therefore, the daily image guidance has limited value to improve the collective mean dose delivery accuracy among a patient population, but it is more valuable in preventing large dosimetric errors for a small percentage of patients who have more systematic and random set up error. Because of the lack of predictive parameters to differential patient set up quality before treatment, daily MVCT imaging guidance is considered a preferable step and important safety measure in head and neck cancer IMRT treatment.

**List of tables and figures:**

Table 1. Characteristics of 10 nasal cavity and nasopharyngeal cancer patients.

Table 2. Mean value of daily set up error for nasal cavity and nasopharyngeal cancer patients using MVCT co-registration to initial planning kVCT

Table 3. EUD *a* optimization parameters for head-and-neck target and normal structures

Table 4. The EUD change for OARs caused by daily positioning error. Units in Gy.

Figure 1. A typical DVH comparison of selected organs for a patient in 3 scenarios: (a) daily repositioned with MVCT co-registration with original planning CT, (b) with only weekly MVCT imaging guidance and (c) without imaging guidance at all. Comparing with scenario (a), (c) shows slightly degraded dosimetry with more under-dosed area in the PTV and over dose area in most OARs. Scenario (b) falls in between (a) and (c).

Figure 2. The lowered PTV EUD due to the lack of daily image guidance. (a) the absolute values of EUD; (b) The normalized value of EUD.

Figure 3. The vertical daily adjustment with MVCT of a patient shows loose linear trend towards one direction.

Figure 4. The normal distribution of the spinal cord dose error from the treatment plan without or with weekly imaging guidance.

**References**

Lee N, XP, Quivey JM, Sultanem K, et al, Intensity-modulated radiotherapy in the treatment of nasopharyngeal carcinoma: an update of the UCSF experience*.* *Int J Radiat Oncol Biol Phys.* 2002. **53**:12-22.

Nutting C, Dearnaley DP, and Webb S, Intensity-modulated radiation therapy: A clinical review. *Br J Radiol* 2000. **73**:459–469.

Eisbruch A, Ten Haken RK, Kim HM *et al.*, Dose, volume, and function relationships in parotid salivary glands following conformal and intensity-modulated irradiation of head and neck cancer. *Int J Radiat Oncol Biol Phys* 1999. **45**:577–587.

Xia P, Fu KK, Wong GW *et al.*, Comparison of treatment plans involving intensity modulated radiotherapy for nasopharyngeal carcinoma. *Int J Radiat Oncol Biol Phys*. 2000. **48**:329–337.

Hunt MA, Zelefsky MJ, Wolden S *et al.*, Treatment planning and delivery of intensity-modulated radiation therapy for primary nasopharynx cancer. *Int J Radiat Oncol Biol Phys*. 2000. **49:**623–632.

Sultanem K, Shu HK, Xia P *et al.*, Three-dimensional intensity-modulated radiotherapy in the treatment of nasopharyngeal carcinoma: The University of California-San Francisco experience. *Int J Radiat Oncol Biol Phys.* 2000. **48**:711–722.

Gilbeau L, Octave-Prignot M, Loncol T, *et al*. Comparison of set up accuracy of three different thermoplastic masks for the treatment of brain and head and neck tumors. *Radiother Oncol.* 2001. **58**:155-162.

Hurkmans CW, Remeijer P, Lebesque JV *et al.*, Set-up verification using portal imaging; review of current clinical practice, *Radiother Oncol.* 2001. **58**:105–120.

Hong TS, Tomé WA, Chappell RJ, *et al*. The impact of daily set up variations on head-and-neck intensity-modulated radiation therapy. *Int J Radiat Oncol Biol Phys.* 2005. **61**:779-788.

van Herk M, Remeijer P, Lebesque JV, "Inclusion of geometrical uncertainties in treatment plan evaluation," *Int J Radiat Oncol.* 2001. **52**:1400-1409.

Manning MA, Wu Q, Cardinale RM, *et al*. The effect of set up uncertainty on normal tissue sparing with IMRT for head-and neck cancer. *Int J Radiat Biol Phys.* 2001. **51**:1400-1409.

Beckham WA, Keall PJ, Diebers JV. A fluence-convolution method to calculate radiation therapy dose distributions that incorporate random set-up error. *Phys Med Biol.* 2002. **47**:3465-3473.

Ploquin N, Song W, Lau H *et al*. Intensity modulated radiation therapy for oroharyngeal cancer: the sensitivity of plan objectives and constraints to set-up uncertainty. *Phys Med Biol.* 2005. **50**:3515-3533.

Sheng K., Molloy J., Read P. IMRT Dosimetry of the head and neck: A Comparison of Treatment Plans Using Linac-Based IMRT and Helical Tomotherapy. *Int. J. Radiat. Oncol. Biol.2006 Phys.***65**:3; 917-923.

Sheng K, Larner J, Molloy J, Read P**.** dosimetric comparison of non-coplanar IMRT versus Helical Tomotherapy for nasal cavity and paranasal sinuscancer.Radiother Oncol. 2007 Feb;**82**(2):174-8.

Yan, D., et al., *Adaptive modification of treatment planning to minimize the deleterious effects of treatment set up errors.* Int J Radiat Oncol Biol Phys, 1997. **38**(1): p. 197-206.

Xing L, Lin Z, Donaldson SS, *et al*. Dosimetric effects of patient displacement and collimator and gantry angle misalignment on intensity modulated radiation therapy. *Radiother Oncol*. 2000. **56**:97-108.

Niemierko A. Reporting and analyzing dose distributions: A concept of equivalent uniform dose. *Med Phys.* 1997. **24**:103-110.

Wu Q, Mohan R, Niemierko A, *et al*. Optimization of intensity-modulated radiotherapy plans based on the equivalent uniform dose. *Int J Radiat Oncol Biol Phys.* 2002. **52**:224-235.

1. Boswell S., T.W., Jeraj R., Jaradt H., Mackie TR., *Automatic registration of megavoltage to kilovoltage CT images in helical tomotherapy: An evaluation of the set up verification process for the special case of a rigid head phantom.* Medical Physics. 2006. **33**(11): p. 4395-4404.
2. [Li JG, Xing L.](http://www.ncbi.nlm.nih.gov/entrez/query.fcgi?db=pubmed&cmd=Retrieve&dopt=AbstractPlus&list_uids=10947260&query_hl=3&itool=pubmed_docsum) Inverse planning incorporating organ motion. Med Phys. 2000 Jul;**27**(7):1573-8.

22. Thevenaz P. and Unser M. A pyramid approach to sub-pixel image fusion based on mutual information, Proc. IEEE Int. Conf. on Image Processing, Lausanne, Switzerland, September 16-19, 1996, vol. I, pp. 265-268.
